# Differentials in health-related quality of life of employed and unemployed women with normal vaginal delivery

**DOI:** 10.1186/s12905-017-0481-0

**Published:** 2018-01-10

**Authors:** Anthonia U. Chinweuba, Ijeoma L. Okoronkwo, Agnes N. Anarado, Noreen E. Agbapuonwu, Ngozi P. Ogbonnaya, Chikaodili N. Ihudiebube-Splendor

**Affiliations:** 1 University of Nigeria, Nsukka, Enugu State Nigeria; 2 Nnamdi Azikiwe University, Awka, Anambra State Nigeria

**Keywords:** Child care, Education, Employment, Normal vaginal delivery, Postpartum women, Quality of life

## Abstract

**Background:**

The combination of child care and domestic work demands on both housewives and the employed (hired) women may impact their health-related quality-of-life. There is paucity of studies to ascertain this. This study investigated the differences in health-related quality of life of employed and unemployed women with normal vaginal delivery and associated socio-demographic variables.

**Methods:**

This longitudinal study was done from March, 2012 to June, 2013. Modified SF-36v2™ health-related quality of life questionnaire was administered to 234 newly delivered women drawn from six selected hospitals in Enugu, Southeast Nigeria at 6, 12 and 18 weeks postpartum. Respondents were reached for data collection through personal contacts initially at the hospitals of delivery, and subsequently by visits to their homes/workplaces or cell-phone calls. Women were asked to indicate how each of 36 items applied to them at each of the three times. Data collection lasted for six calendar months and 17 days (from September 3rd 2012 to 20th March, 2013).

**Results:**

All the women had their best HrQoL at 12 weeks postpartum. Employed women reported lower health-related quality-of-life than the unemployed at the three time-points, the lowest mean score being at 18 weeks postpartum (Mean = 73.9). Multiple comparison of scores of the two groups using Tukey HSD Repeated Mean showed significant variation on the eight subscales of the health-related quality-of-life. Physical functioning (*p* = 0.045), Physical role limitation (*p* = 0.000), bodily pain (p = 0.000), social functioning (p = 0.000) and general health (p = 0.000) were unequal guaranteeing type 1 error. Women with higher education and personal income reported higher health-related quality-of-life (*p* < 0.05). Employed women have more problems with physical health components and are more negatively affected by increasing age except those with higher education and personal income.

**Conclusions:**

Increased responsibilities combined with increasing age and low socio-economic status reduce women’s health-related quality-of-life post-partum. The traditionally accepted paid 3 months maternity leave should be elongated by extra months to help women balance their daily work with baby care. Gender sensitive employment opportunities in favour of women are necessary to empower more women economically.

**Electronic supplementary material:**

The online version of this article (10.1186/s12905-017-0481-0) contains supplementary material, which is available to authorized users.

## Background

The combination of child care and domestic work demands may impact their health-related quality-of-life (HrQoL) of housewives and the employed (hired) women. HrQoL is self-report of one’s perceived feeling of comfort, ability to realize his/her life potentials and satisfaction with life as expressed in the person’s physical, emotional and social functions of life. This study is a self-report, based on scores obtained on the eight sub-scales and single items of modified Short Form 36 version 2 (SF36v2™) HrQoL instrument by Ware and Sherbourne [1] that includes general health, physical functioning, physical role limitation, emotional role limitation, social functioning, bodily pains, vitality, and mental health of employed and unemployed women. Most available literatures on HrQoL view it as a framework for examining disease and its impact on a patient [1–4]. However, other life situations than disease, such as child birth and care, have been noted to impact on ones HrQoL.The few available studies that compare QoL in newly delivered women with paid job and the unemployed show that the later are often financially dependent and as a result experience more economic hardship than the former [5]. Meanwhile, based on Udo’s [6] report, as at first quarter of 2016, only about 63.8% of Nigerian women were employed – 14% were unemployed while 22.2% were underemployed.

Age was shown by some studies as a strong factor influencing [7–11]. Regidor et al. [11] observes that persons aged 25–44 years had better mental health and general health while those aged 45–64 years had better physical functioning and general health. However, In contrast, Bolton et al. [8] observed that younger adults had higher HrQoL with physical activity than their older counterparts. Socio-economic status (SES) studies showed consistently high HrQoL for the most affluent and the most highly educated subjects, the contrary for the middle and lower income and education groups [12, 13]. A multicentre cross-sectional observational study on SES and HrQoL of 4574 patients with chronic obstructive pulmonary disease by Meravitlles et al. [14] showed similar result.

The purpose of this study is to establish the differences in the eight sub-scales of HrQoL of employed and unemployed women at 6, 12 and 18 weeks postpartum and to determine personal factors of age, education, and personal income (amount of money the woman had direct access to and control of - earned from employer (for employed women) or received as monthly stipend from spouse (for the unemployed)) that influence them, where they exist. This study hypothesized that: there is no difference in the HrQoL of employed and unemployed women following their return to routine tasks along with baby care at 12 and 18 weeks after normal delivery; there is no significant difference in HrQoL scores of employed and unemployed women at 18 weeks postpartum based on their age, education, and personal income. The study is expected to yield self-perception data source for establishing the QoL of postpartum mothers in this part of the world where women are exposed to a lot of socio-economic challenges [15, 16]. Findings may be used as basis for making pertinent recommendations for health policies and legislation on interventions to improve situations of the vulnerable groups and guide the development of strategic plans for health and employment.

## Methods

### Design

This was a longitudinal, prospective descriptive study design as consenting newly delivered mothers were assessed without any intervention to determine their HrQoL changes at three points as they returned to pre-pregnancy daily activities.

### Setting

The study was carried out in three local government areas in Enugu capital territory, f Enugu State, southeastern Nigeria from March, 2012 to June, 2013. Enugu is a city with an area of about 100 km [2]. It encompasses major high density residential and commercial areas in the urban part of Enugu State with a population of up to 100,000 residents [17] and ten residential layouts. *Igbo* language is the people’s vernacular, However, as an urban community, English Language, which is Nigeria’s official language, is spoken and understood by most of the people.

Located in the city are numerous private and public hospitals that function as peripheral and/or referral centres for maternal, newborn and child healthcare services.

### Population of the study

The average monthly registration for 6 weeks post-natal visit in six selected hospitals popular in obstetric health services in Enugu was used to estimate the population of 363 newly delivered women used for the study.

### Sample

Power analysis was used to estimate the sample size of 234 newly delivered mothers. Using the Creative Research Systems survey software of the Sample size calculator formula of: ss = (Z [2]*(p)*(1-p))/C [2]; where: Z = 1.96, p = proportion of target population (estimated to have complication-free normal vaginal delivery) (expressed as 0.5), C = Confidence Interval (.04 ± 4) [18], a sample of 187 was initially estimated. Applying the adjusted sample size formula for anticipated 20% attrition rate: q = n/1-f (where q is adjusted sample size; n is original sample size; and, f is estimated non-response rate) [19], the initial sample size estimate was adjusted from 187 to 234.. This sample size represented about 64.5% of the population.

Multi-stage sampling technique was adopted in the process of selecting the sampling units. Firstly, the city was stratified using its three Local Government Areas (LGAs). Then, two health facilities from each LGA commonly used by child bearing women were purposively selected (a total of 6 health facilities). The sample units to be recruited were proportionately predetermined to be 50 % (117) employed and unemployed women, respectively.

The women that met the following inclusion criteria were recruited into the study: women aged between 20 and 44 years; had normal delivery of a healthy term baby in any of the selected hospitals within the past 6 weeks at the time of selection; either on a paid job outside home or an unpaid housewife; without history of complicated pregnancy/medical conditions; no stress-inducing experiences (such as loss of a family member, divorce, or family problems); and, women who consent to participate. Consent of spouses of women who opted to be visited at home for the second and third rounds of data collection at 12 and 18 weeks post delivery respectively, were obtained to allow researcher to visit their matrimonial home.

### Instrument

Modified form of the standardized Iranian version of Short-Form 36-item Version 2 Health Survey Questionnaire (SF-36v2™) was used for data collection in this study. The SF-36v2™ is a standardized popular generic short form of HrQoL survey instrument developed by Ware and Sherbourne in 1996 with 36-item self-rated health status profile [20–24]. Iranian version of the SF-36v2™ is also standardized [22]. It measures eight health-related concepts: physical functioning (limitations in typical daily physical activities due to health problems), physical role limitation (limitations with work or other regular daily activities due to physical health problems), bodily pain, general health perceptions (self-rated health), vitality, social functioning (limitations in social activities with the family, friends, neighbours, or groups such as visits, meetings, wedding and burial ceremonies), emotional role limitation (limitations with work or other regular daily activities as a result of any emotional problems, such as feeling depressed or anxious), and perceived mental health (how the woman feels and how things have been with her). Psychometrically, these eight scales are hypothesized to form two distinct higher-order clusters – physical health component summary (PCS) and mental health component summary (MCS) – since they have certain physical and mental health variance in common. Thus, the taxonomy of items and concepts underlying the construction of the SF-36 scales and summary measures are at three levels, namely: 36 items; eight sub-scales that aggregate 2–10 items each; and, two summary measures that aggregate sub-scales.

The instrument is validated to be a self-completed or as interviewer-administered questionnaire in English Language under the name of SF-36v2™^1^. Montazeri et al. [22] did a translation and validation study of the Iranian version of this SF-36v2™. The SF-36v2™ has been used worldwide in many studies [1, 4, 13, 22, 25, 26] and the Iranian version was recorded to have a well documented validity [22]. Both MCS and PCS of this Iranian version were shown to have satisfactory internal consistency reliabilities. Cronbach’s alpha coefficient for the PCS was 0.87 and 0.82 for the MCS.

The English version of the instrument was used as this is the common language understood and spoken by all members of the study group. However, for clarity and better understanding of terms by respondents in the study area, some language modifications were made in some items of the original standardized Iranian version SF-36v2™ as attached [see Additional file 1]. Also, 3-item questionnaire was used to collect data on respondents’ demographic information on age, educational level, and personal income per month. Education was based on highest educational qualification attained. For the purposes of reporting HrQoL by personal income, women were categorised into four groups based initially on Nigeria’s minimum wage of N18,000.00 (US$107) per month for government-employed workers and subsequently, by equivalent personal income (in equivalent sizes of N50,000.00 (US$298) per month (between groups). For every woman recruited for possible inclusion, personal data including full name, popular (or preferred) name, home address, office address (where applicable), phone number (where available), and preferred contact mode (visit at home or office) were documented.

The researcher-modified instrument was validated for context and content by presenting a draft of it and a copy of the original instrument, as well as the research objectives, and scope to a professor in Measurement and Evaluation to comment on the appropriateness of arrangements and the modifications. To ascertain its reliability, 20 copies were administered to 20 postnatal women with similar characteristics that attended a specialist maternity hospital in another state in eastern Nigeria. The percentage scores of responses for each section of the instrument were subjected to split-half reliability test using Pearson’s Product Moment Correlation. The alpha coefficient result of 0.87 was considered satisfactory.

### Data collection

First contact with the women was when they visited health facility for 6 weeks postpartum care. Following self-introduction of researcher (or assistant) and establishment of rapport, purpose of the study and need for repeat visits were explained, while necessary history and consent to participate were sought. Upon accepting to participate, and after signing the informed consent sheet, those who met the inclusion criteria had a copy of the instrument administered to them. They were asked to read the contents as carefully as possible and respond to each item as it applied to them. For those who could not read and/or write (due to their low level of education), the researcher or assistant helped by reading out the contents to the respondents and filling in the responses as objectively as possible. After 6 weeks, the researcher and/or assistant visited each respondent at the preferred contact place for second round data collection. For those who preferred interview through phone call, data were collected through cell phone discussion guided strictly by contents of the instrument. At the third round, data collection was repeated after another 6 weeks through the respondent-preferred means. Employed women who could not resume work when due (after 12 weeks), for any reason, were removed from the study. Data collection lasted for six calendar months and 17 days (from September 3rd 2012 to 20th March, 2013).

### Method of data analysis

Data were analysed descriptively using frequency, percentages, mean and standard deviation (SD). For each scale of the SF-36v2™ the mean was computed and scores converted to percentages to ensure true proportion of values. The scores at 6, 12 and 18 weeks were subjected to chi square test to determine the differences in dimensions of HrQoL for the employed and unemployed. Tukey test was used to compare the mean of the women’s HrQoL scores at 6, 12 and 18 weeks postpartum while the data were subjected to analysis of variance (ANOVA) to determine the interaction between the two groups of women and their demographic variables of age, educational level and personal income at 6, 12 and 18 weeks postpartum. All statistical analyses were performed using the IBM SPSS statistical software package version 23. All values of *p* < 0.05 were considered significant.

## Results

Of the 234 women enrolled at baseline, 23 (9.8%) (10 unemployed and 13 employed women) were lost due to voluntary withdrawal, incorrect address/phone number, loss of baby, or failure to resume work at 12 weeks post delivery. Three unemployed women were deliberately withdrawn to equalize number with employed women. Final sample was 208 women (104 employed and 104 unemployed women), 88.9% of the baseline sample [see Additional file 2: Figure 1].

Table 1 showed the mean (±SD) age of participants as 27.91(±5.89) years, comprising mainly women within the age range of 20–24 years (36.0%) and 25–29 years (30.8%) respectively. While all the women, except 2 unemployed, had some form of formal education, more of the employed women (53 _=_ 51.0%) than the unemployed (16 = 15.4%) had a tertiary education. The mean personal income per month for the participants was N48,159.00. The unemployed earned average of N27,087.00, while the employed earned higher (N69,231.00). Twenty-one out of 33 women that earned above N100,000.00 were employed. Conversely, 48 out of 52 women that earned less than N18,000.00 (Nigeria minimum wage standard) were unemployed.

**Table 1:** Characteristics of the respondents

		Total (*n* = 208)		Unemployed (104)		Employed (104)	
		F	%	F	%	F	%
Age	20–24	75	36.0	43	41.3	32	30.8
	25–29	64	30.8	34	32.7	30	28.8
	30–34	37	17.8	18	17.3	19	18.3
	35–39	21	10.1	8	7.7	13	12.5
	40–44	11	5.3	1	1.0	10	9.6
	Mean(SD)	27.91(5.89)		26.71(4.97)		29.07(6.52)	
Education	No formal	2	1.0	2	1.9	0	0
	Primary	24	11.5	21	20.2	3	2.9
	Secondary	113	54.3	65	62.5	48	46.1
	Higher (Tertiary)	69	33.2	16	15.4	53	51.0
Personal income	Less than N18,000	52	25.0	48	46.15	4	3.8
	N18.000- N50,000	95	45.7	48	46.15	47	45.2
	N51,000-N100,000	38	18.3	6	5.8	32	30.8
	> N100,000	23	11.0	2	1.9	21	20.2
	Mean	N48,159		N27,087		N69,231	

Repeated measures ANOVA of scores on the eight subscale showed that both groups of women had their best HrQoL at 12 weeks postpartum. In all the sub-scales, the employed women reported lower HrQoL at the three time-points than the unemployed, the poorest being at 18 weeks postpartum (Mean = 73.9). At 18 weeks, the employed women reported more bodily pain, poorer physical role functioning and poorer social functioning (Mean scores: 51.0; 51.2; 52.9 respectively) than the unemployed (mean scores: 84.5; 88.3; 88.4 respectively). There were significant mean score differences between the unemployed and the employed women in the HrQoL subscales of physical functioning (*p* = 0.045), physical role limitation (*p* = 0.000), bodily pain (p = 0.000), general health (p = 0.000), and Social functioning (p = 0.000) over time. (Table 2).

**Table 2:** ANOVA of scores on eight subscales of HrQoL for the employed and unemployed women at 6, 12 and 18 weeks using Tukey Test

HrQoL Subscale		Mean		Std. Error	Sum of Squares	F	Sig.	Univariate Tests Sig
	Time	U	E					
Physical functioning	6 wks	93.5	92.3	.950	2621.520	18.024	.000	.045
	12 wks	98.2	96.0	.839	786.950	5.010	.001	
	18 wks	95.8	93.9	.937	974.729	5.811	.000	
Physical role limitation	6 wks	80.6	75.2	.697	428.260	3.500	.009	.000
	12 wks	93.1	90.2	.918	1001.184	6.571	.000	
	18 wks	88.3	51.2	1.186	5743.328	3.690	.006	
Bodily pain	6 wks	81.5	80.1	1.287	512.099	1.238	.296	.000
	12 wks	85.5	89.6	1.618	3925.263	7.689	.000	
	18 wks	84.5	51.0	1.085	4297.025	3.412	.010	
General health	6 wks	91.6	86.7	.932	804.184	4.109	.003	.000
	12 wks	94.3	89.4	.700	2445.029	26.413	.000	
	18 wks	89.3	85.3	1.228	9117.891	46.166	.000	
Vitality	6 wks	82.4	83.1	1.496	1691.671	3.722	.006	.816
	12 wks	93.2	88.9	1.123	7856.645	39.611	.000	
	18 wks	85.0	87.6	1.267	2483.240	8.569	.000	
Social functioning	6 wks	84.5	84.8	1.301	1626.616	5.442	.000	.000
	12 wks	88.4	86.8	1.339	4386.555	13.377	.000	
	18 wks	88.4	52.9	1.087	4063.882	2.839	.025	
Emotional role limitation	6 wks	81.7	80.5	1.515	1423.269	3.274	.013	.223
	12 wks	88.5	94.1	1.012	2421.128	11.992	.000	
	18 wks	93.9	81.9	.633	1847.427	9.648	.000	
Mental health	6 wks	85.4	87.1	1.339	1949.560	6.054	.000	.590
	12 wks	88.6	88.2	1.270	3247.683	11.030	.000	
	18 wks	87.0	87.5	1.260	1969.339	6.396	.000	

NB: *U* Unemployed, *E* Employed

Degree of freedom for all subscales = 4

Result of multiple comparison of scores of the employed and unemployed women in different age groups using Tukey HSD Repeated Mean showed that the eight subscales of the HrQoL varied significantly. However, physical role limitation, bodily pain and social functioning were unequal guaranteeing type 1 error. Young women (aged 20–25 years), however, had the lowest scores on mental health (Table 3).

**Table 3:** Tukey HSD Repeated Mean comparison of the HrQoL subscales of the women based on their age groups

HrQoL subscale	Age					Sig		
	20–24 yrs. (75)	25-29 yrs. (64)	30-34 yrs. (37)	35-39 yrs. (21)	40-44 yrs. (11)	Time 1	Time 2	Time 3
Physical functioning	95.10	95.10	94.14	93.62	91.32	.182	.776	.094
Physical role limitation	82.02	80.45	80.18	77.40	70.23	1.000	.191	–
Bodily pain	79.99	79.53	78.18	78.13	74.38	.146	–	–
General health	91.57	90.94	89.40	85.18	80.74	1.000	1.000	.085
Vitality	90.06	89.01	87.14	86.01	82.93	.147	.165	.187
Social functioning	84.42	82.63	79.29	78.79	73.65	.086	.087	–
Emotional role limitation	88.96	86.86	86.43	84.07	81.47	.102	.066	.119
Mental health	90.69	89.76	89.14	84.77	83.78	.976	.068	.883

NB: Harmonic Mean Sample Size = 25.705

The mean scores of the employed and unemployed women in different age group categories when compared using ANOVA showed that the mean score differences in the eight subscales of the HrQoL within the population sampled were highly significant (*p* < 0.05) at 95% confidence interval. Table 4.

**Table 4:** ANOVA of respondents’ mean scores on eight subscales of HrQoL at 6, 12 and 18 weeks based on the women’s age groups

Age (years)	20–24		25–29		30–34		35–39		40–44		F	Sig
	x (43)	Y 32)	x (34)	Y (30)	x (18)	y (19)	x (8)	y (13)	x (1)	y (10)		
Physical functioning	71.1	72.7	78.2	73.2	85.6	73.1	100	91.0	82.0	86.6	7.809	.000
Physical role limitation	62.9	67.2	75.3	51.8	93.3	47.1	98.7	74.6	70.0	31.0	7.388	.000
Bodily pain	58.2	42.6	73.3	55.8	72.8	47.1	86.9	54.6	55.0	29.5	14.786	.000
General health	73.9	63.3	75.5	72.0	84.4	80.4	97.9	79.7	83.3	77.7	29.314	.000
Vitality	74.4	64.1	73.8	82.6	80.0	81.0	96.2	79.2	90.0	85.0	8.413	.000
Social functioning	53.7	48.4	76.7	57.0	73.3	53.6	100	62.3	80.0	23.0	7.969	.000
Emotional role limitation	68.8	61.7	74.5	69.3	93.3	67.5	91.7	76.9	80.0	81.3	14.347	.000
Mental health	68.2	65.9	74.8	87.5	70.0	85.8	98.1	85.8	90.0	85.5	4.699	.003

NB:x = Unemployed women; y = Employed women

Degree of freedom for all subscales = 3

Multiple comparisons of the mean scores of the employed and unemployed women in categories of educational groups using Tukey HSD Repeated Mean test (Table 5.) showed that the variance in the eight sub scales of the HrQoL were significant. The variations were less for vitality, social functioning and mental health but were unequal guaranteeing type 1 error.

**Table 5:** Tukey HSD Repeated Mean comparison of HrQoL subscales scores of the women based on their educational level

HrQoL subscales	No formal education (2)	Primary education (24)	Secondary education (113)	Higher education (69)	Sig (α = 0.05)		
					Time 1	Time 2	Time 3
Physical functioning	82.17	84.35	86.70	89.53	.134	–	–
Physical role limitation	82.67	81.71	82.03	78.96	.839	–	–
Bodily pain	74.67	76.88	80.54	76.49	.569	–	–
General health	88.50	89.86	90.83	87.09	.242	–	–
Vitality	86.83	80.31	87.13	88.34	.066	.947	–
Social functioning	82.83	91.22	95.49	94.13	1.000	.268	–
Emotional role limitation	88.17	85.63	88.03	85.11	.479	–	–
Mental health	90.00	86.83	81.24	75.66	.458	.099	–

NB: Harmonic Mean Sample Size = 7.080

The mean scores of the employed and unemployed women in different c educational levels when compared using ANOVA showed that the variance in the eight subscales of the HrQoL within the population sampled were highly significant (*p* < 0.05) at 95% confidence interval except for social functioning. Table 6.

**Table 6:** ANOVA of respondents’ mean of scores on eight subscales of HrQoL at 6, 12 and 18 weeks based on the women’s educational level

Educational level	Unemployed				Employed				F	Sig
	N (2)	P (21)	S (65)	H (16)	N (0)	P (3)	S (48)	H (53)		
Physical functioning	60.1	75.4	85.5	92.7	–	61.9	86.3	95.5	11.080	.000
Physical role limitation	48.7	65.0	75.0	94.1	–	55.5	62.0	93.3	18.260	.000
Bodily pain	51.5	58.3	70.0	83.7	–	58.4	55.7	88.3	3.796	.011
General health	66.2	60.1	78.3	71.9	–	65.1	65.7	88.9	14.329	.000
Vitality	61.2	49.0	75.0	94.6	–	75.6	66.8	90.0	14.772	.000
Social functioning	68.8	59.5	70.0	90.9	–	57.5	51.4	91.6	2.013	.113
Emotional role limitation	58.3	65.5	80.0	74.6	–	57.0	75.0	88.9	8.406	.000
Mental health	59.7	54.0	77.5	73.0	–	71.7	53.6	95.8	5.309	.002

NB: *N* No formal education, *P* Primary, *S* Secondary, *H* Higher

Std Dev = Standard Deviation; Degree of freedom for all subscales = 3

The mean scores of the employed and unemployed women in different categories of personal when compared using Tukey HSD Repeated Mean test (Table 7) showed that the eight subscales of the HrQoL had significant variation. Unequal variations were noted for physical functioning, bodily pain vitality and mental health guaranteeing type 1 error.

**Table 7:** Tukey HSD Repeated Mean comparison of HrQoL subscales scores of the women based on their personal income

HrQoL Subscales	<N18,000 (52)	N18,000-N50,000 (95)	N51,000-N100,000 (38)	>100,000 (23)	Sig (α = 0.05)		
					Time 1	Time 2	Time 3
Physical functioning	92.49	96.03	92.85	94.79	.101	.604	–
Physical role limitation	86.89	80.17	75.91	71.56	1.000	1.000	1.000
Bodily pain	81.16	79.44	75.70	75.15	.110	.801	–
General health	90.83	90.72	88.52	82.67	1.000	1.000	.999
Vitality	84.57	87.00	89.69	85.67	.191	.122	–
Social functioning	83.01	82.33	78.03	75.72	.578	.084	.981
Emotional role limitation	88.19	86.86	85.83	82.38	1.000	.629	.404
Mental health	84.20	87.74	88.56	90.63	.060	.172	–
Harmonic Mean Sample Size			40.18				

The mean scores of the employed and unemployed women in different categories of personal income when compared using ANOVA showed that the variance in the eight subscales of the HrQoL within the population sampled were highly significant (p < 0.05) at 95% confidence interval. Table 8.

**Table 8:** ANOVA of respondents’ mean scores on the eight subscales of HrQoL at 6, 12 and 18 weeks based on the women’s personal income

Personal income (N)/month	<N18,000		N18,000 – N50,000		N51,000 -N100,000		>N100,000		F	Sig
	x (48)	y (4)	x (48)	y (47)	x (6)	y (32)	x (2)	y (21)		
Physical functioning	86.0	61.9	91	84.4	100	89.2	96.3	89.2	8.893	.000
Physical role limitation	67.5	54.7	80.0	44.4	100	60.8	92.5	60.8	38.275	.000
Bodily pain	82.5	60.2	66.7	40.9	88.7	66.2	91.2	66.2	4.607	.004
General health	81.7	78.3	83.3	83.3	95.2	83.3	94.1	83.3	42.578	.000
Vitality	60.0	69.5	83.3	82.2	97.5	85.7	72.0	85.7	6.850	.000
Social functioning	75.0	49.0	76.6	48.8	98.7	62.3	65.4	62.3	6.891	.000
Emotional role limitation	66.7	62.8	77.8	68.8	98.3	76.5	77.8	76.5	15.133	.000
Mental health	62.5	60.5	83.3	80.9	93.7	87.9	71.0	87.9	7.006	.000

NB:x = Unemployed women; y = Employed women; Std Dev. = Standard Deviation

Degree of freedom for all subscales = 3

## Discussion

A major finding from this study was that quality-of-life of post-natal women was best at 12 weeks but dropped as they resumed work outside the home. This implies that newly delivered women had the best HrQoL within their maternity leave period. Women who return to work after 12 weeks maternity leave may find their job more challenging with additional task of baby care particularly women without significant social support for domestic tasks. This increased responsibility and workload will most likely affects their HrQoL trajectories negatively. This suggests the need for a domestic social support, longer period of maternity leave and reduced workload, particularly for the employed women.

Bodily pains and problems with physical health reported by the women 6 weeks after resumption of work were likely to negatively affect their job performance. Their social functions might also be compromised. The findings, thus, different from Greenberg’s [5]. Conflict between the women’s formal or informal jobs and domestic tasks may make routine tasks cumbersome to accomplish. Poor social functioning may lead to depression, withdrawal, loss of concentration and aggressive behaviours. Since increased task, and reduced physiological or psychological energy result in less activity tolerance, their productivity in workplace is also likely to reduce.

Increasing age coupled with increasing responsibility could reduce one’s HrQoL. Findings from this study indicated that with increasing age, the women, especially the employed, experienced more bodily pains, which may have affected their social functions and regular daily activities. This finding was consistent with those of Cohen [7]; Bolton [8], Bonsergent [9], and Al-Farsi [10]. The older women were likely to have a higher parity. This and similar social and cultural factors may mean increased responsibilities and increased demand for physical energy and social involvements. Unemployed and employed women aged 20–25 years had the lowest scores on mental health probably because being young the women are likely to be inexperienced mothers. Lack of experience can be a source of frustration and stress as the woman still undergoes some motherhood adaptation processes. It is not clear, however, why the unemployed women aged 25–35 years had poorer mental health than the employed women of the same age. Further studies are recommended for this.

The higher the women’s educational level, the higher their reported HrQoL irrespective of their employment status. Highly educated women have increased chance of getting well-paid job which in turn increases their affordability and accessibility to quality living conditions – this is probably independent of their current employment status. This was in agreement with earlier studies such as Ross et al. [12] and Meravitlles et al. [14]. However, unemployed women with tertiary education had reduced scores on the subscales of general health, emotional role limitation and mental health when compared with their counterparts. It is possible therefore that high educational attainment without attendant practice reduces ones emotional and mental health. If this is so, then, it will most likely be prominent among women who become housewives not by their own volition but as a result of some external factors such as spouse disapproval. Being a housewife but restricted from formal work despite educational attainment may, therefore, affect the woman’s psyche which may be manifested in poor physical and mental health.

HrQoL of the women increased with their personal income irrespective of time. High personal income increases access to comfort and enhances psycho-social wellbeing. This implies a better state of mind and body. Although more employed women (21) than the unemployed (2) that entered the study had monthly personal income above N100,000.00, it is significant to observe that women with higher personal income, whether unemployed or employed, had higher scores on the HrQoL scales. Studies such as Ross et al. [12] and Tucker [13] support this positive correlation of HrQoL with SES. The findings suggest SES as a measure of health and that employed women, particularly those with higher education that increases their chances of better placement on the job, had more satisfactory life. This is supported by Meravitlles et al. [14] and Tucker et al. [13]. With evidence from the findings, the ability to have reasonable access to income is essentially a means to an improved HrQoL.

A major strength of this study is its prospective design and multiple time evaluation of the women’s HrQol. which enabled the researchers to identify HrQol change overtime. As a limitation, the study was conducted in an urban area where most women had access to good quality living conditions. Rural dwellers live with higher level of poverty than urban dwellers. The findings therefore cannot be extended to employed and unemployed women in rural settings. The respondents were constrained by the scales; they did not have the opportunity to express other ideas outside of the items in the SF-36v2™. Further qualitative studies are indicated to elicit some socio-cultural factors and issues in postpartum HrQoL of Nigerian women.

## Conclusion

The study revealed that newly delivered women’s HrQoL is at its peak at 12 weeks postpartum and starts to decline as they go back to their routine full time job, especially for the employed women who reported more problems with physical health components. Employed women are more negatively affected by increasing age except those with higher education and personal income.

It is recommended that the traditionally accepted paid maternity leave of 3 months should be elongated by extra months to help the woman balance her daily work with baby care demands while she maintains a good HrQoL. However, further research is required to determine the best duration for maternity leave. Also, paternity leave should be granted to fathers, on request, so that they will have time at home to assist their spouse as may be necessary. Gender sensitive employment opportunities should be created to empower more women economically. Programmes should be designed to ensure that these at risk groups, that is women of child bearing age, earn at least the national minimum wage of N18,000.00 to enhance their economic status.

## Additional files

Additional file 1. Instrument modification: Details of modifications on specified items of the original standardized Iranian English version SF-36v2™. (DOCX 14 KB)

Additional file 2: Figure 1. Flow chart indicating attrition of women from the study. (PPTX 43 KB)

Additional file 3. Instrument: Sample of instrument used to collect data on personal profile of respondents and the modified SF-36v2™. (DOCX 34 KB)

## Abbreviations

HrQoL: Health-related Quality of Life
LGA: Local Government Area
MCS: Mental Component Summary
PCS: Physical Component Summary
QoL: Quality of Life
SES: Socio-Economic Status
SF-36v2™: Short-Form 36-item Version 2 Health Survey Questionnaire
