# Supplementary material for: Differentials in health-related quality of life of employed and unemployed women with normal vaginal delivery
Source: BMC Womens Health. 2018 Jan 10;18:13. doi: 10.1186/s12905-017-0481-0 (PMC5764022; doi:10.1186/s12905-017-0481-0)
Supplement: Supplementary file 1 — Instrument modification: Details of modifications on specified items of the original standardized Iranian English version SF-36v2™. (DOCX 14 KB) [file 12905_2017_481_MOESM1_ESM.docx]

**Instrument Modification**

| **Item** | **MODIFICATION** |
| --- | --- |
| **2** | - Substitute “compared to *one year* ago” with “compared to *six weeks* ago” at second and third visits. |
|  | - Change four (4) weeks (interval between visits) to six (6) weeks |
| **3(b)** | Delete ‘bowling or playing gulf’; Add ‘lifting bucket of water’ |
| **(c)** | Substitute ‘groceries’ with ‘food items from the market’ |
| **(d)** | Add (more than one storey building) |
| **(e)** | Add (first floor of a storey building) |
| **(g)** | Substitute ‘mile’ with ‘kilometre’ |
| **(h)** | Change “Walking several hundred yards” to, “Walking up to half kilometre” |
| **(i)** | Change “Walking one hundred yards” to, “Walking less than half kilometre” |
| **4** | Substitute ‘role physical’ with ‘physical role limitation’ |
| **5** | Substitute ‘role emotional’ with ‘emotional role limitation’ |
| **6** | Instead of “During the past 6 weeks, to what extent has your physical health or emotional problems interfered with your normal social activities with family, friends, neighbours, or groups?”, Ask “During the past 6 weeks, to what extent has your physical health or emotional problems interfered with your normal social activities, that is, your interpersonal relationships with family, friends, neighbours, or groups such as visits, meetings, wedding and burial ceremonies, etc” |
| **9** | Delete ‘Did you have a lot of energy?’  Substitute - ‘pep’ (informal for pepper) with ‘strength’  - ‘down in the dumps’ with ‘sad and hopeless’  - ‘blue’ with ‘depressed’  - ‘been a happy person’ with ‘been happy’  Merge ‘Did you feel worn out?’ with ‘Did you feel tired?’ to have ‘Did you feel tired and worn out?’  Change ‘a little easier’ to ‘a little more readily’  Merge ‘Have you felt so sad and hopeless that nothing could cheer you up?’ and ‘Have you felt downhearted and depressed’ to be: ‘Have you felt so sad, hopeless, down-hearted and depressed that nothing could cheer you up?’ |
| **HrQoL Sub-teams of components indicated after each question to guide the respondent** | |
