# Supplementary material for: Differentials in health-related quality of life of employed and unemployed women with normal vaginal delivery
Source: BMC Womens Health. 2018 Jan 10;18:13. doi: 10.1186/s12905-017-0481-0 (PMC5764022; doi:10.1186/s12905-017-0481-0)
Supplement: Supplementary file 2 — Flow chart indicating attrition of women from the study. (PPTX 43 KB) [file 12905_2017_481_MOESM2_ESM.pptx]

## Slide 1
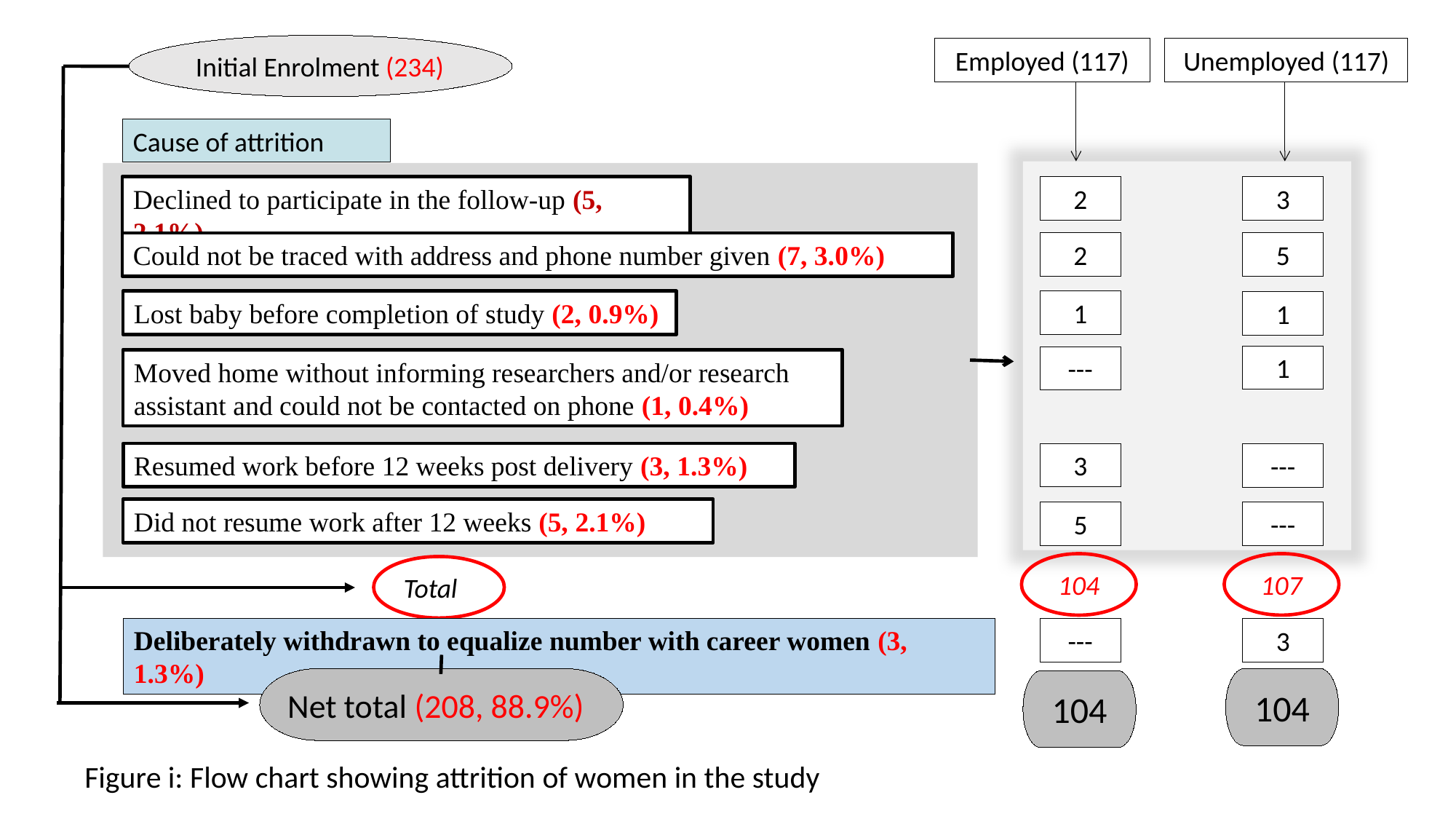

Initial Enrolment (234)
Employed (117)
Unemployed (117)
Cause of attrition
Declined to participate in the follow-up (5, 2.1%)
2
3
Could not be traced with address and phone number given (7, 3.0%)
2
5
Lost baby before completion of study (2, 0.9%)
1
1
1
---
Moved home without informing researchers and/or research assistant and could not be contacted on phone (1, 0.4%)
Resumed work before 12 weeks post delivery (3, 1.3%)
3
---
Did not resume work after 12 weeks (5, 2.1%)
5
---
107
104
Total
Deliberately withdrawn to equalize number with career women (3, 1.3%)
---
3
Net total (208, 88.9%)
104
104
Figure i: Flow chart showing attrition of women in the study
