# Supplementary material for: Differentials in health-related quality of life of employed and unemployed women with normal vaginal delivery
Source: BMC Womens Health. 2018 Jan 10;18:13. doi: 10.1186/s12905-017-0481-0 (PMC5764022; doi:10.1186/s12905-017-0481-0)
Supplement: Supplementary file 3 — Instrument: Sample of instrument used to collect data on personal profile of respondents and the modified SF-36v2™. (DOCX 34 KB) [file 12905_2017_481_MOESM3_ESM.docx]

**INSTRUMENT**

**Instruction:**

This checklist explores the post-delivery quality of life of employed and unemployed women in Nigeria. There are no right or wrong answers, because each woman has her own peculiar life circumstances. Be sure to answer every item. Begin by completing the following:

| **Section A: Personal Profile of Respondent** |
| --- |

**1 Age group (in years)**

20 – 24 25 – 29

30 – 34 35 – 39

≥ 40

**2 Educational status**

No formal education Primary

Secondary Higher/University

**3 Employment**

Unemployed

Employed

**4 Estimated personal income per month**

Less than N18,000.00

N18,000.00 – N50,000.00

N51,000.00 – N100,000.00

> N100,000.00

| **Section B: Modified SF-36v2^TM^** |
| --- |

**Instructions for completing the Questionnaire**

This survey asks for your views about your health. This information will help you keep track of how you feel and how well you are able to do your usual activities. It will help the researcher determine how daily activities affect the health-related quality of life of mothers four months after delivery.

Please answer every question. Some questions may look like others, but each one is different. Please take the time to read and answer each question carefully by filing in the bubble that best represents your response. If you are unsure about how to answer a question, please give the best answer you can.

**EXAMPLE**

**This is for your review.** Do not answer this question. The questionnaire begins with the section ***Your Health in General*** below.

For each question you will be asked to fill in a burble in each line.

1 How strongly do you agree or disagree with each of the following statements?

Strongly Agree Disagree Strongly

Agree disagree

1. I enjoy listening to music ○ ● ○ ○
2. I enjoy reading magazines ● ○ ○ ○

**Please begin answering the questions**

| **Your Health in general** |
| --- |

**1 In general, would you say your health is** *(general health – GH)*

| Excellent | Very good | Good | Fair | Poor |  |
| --- | --- | --- | --- | --- | --- |
| ○ | ○ | ○ | ○ | ○ | GH01 |

**2 Compared to one year ago, how would you rate your health now?**

| Much better now than one year ago | Somewhat better now than one year ago | About the same as one year ago | Somewhat worse now than one year ago | Much worse now than one year ago |  |
| --- | --- | --- | --- | --- | --- |
| ○ | ○ | ○ | ○ | ○ | GH02 |

**3 The following questions are about activities you might do during a typical day. Does your health now limit you in these activities? If so how much?** *(physical functioning – PF)*

|  | Yes, limited  a lot | Yes,  limited  a little | No, not  limited  at all |  |
| --- | --- | --- | --- | --- |
| a. Vigorous activities such as running, lifting heavy objects, participating in strenuous sports | ○ | ○ | ○ | PF01 |
| b. Moderate activities like moving a table, lifting a bucket of water, pushing a vacuum cleaner | ○ | ○ | ○ | PF02 |
| c. Lifting or carrying food items from market | ○ | ○ | ○ | PF03 |
| d. Climbing several flights of stairs (more than one storey building) | ○ | ○ | ○ | PF04 |
| e. Climbing one flight of stairs (first floor of a storey building) | ○ | ○ | ○ | PF05 |
| f. Bending, kneeling or stooping | ○ | ○ | ○ | PF06 |
| g. Walking up to half kilometre | ○ | ○ | ○ | PF07 |
| h. Walking less than half kilometre | ○ | ○ | ○ | PF08 |
| i. Walking one hundred yards | ○ | ○ | ○ | PF09 |
| j. Bathing or dressing yourself | ○ | ○ | ○ | PF10 |

**4 During the past 6 weeks, how much of the time have you had any of the following problems with your work or other regular daily activities as a result of your physical health?** *(physical role limitation – PRL)*

|  | All of the time | Most of the time | Some of the time | A little of the time | None of the time |  |  |
| --- | --- | --- | --- | --- | --- | --- | --- |
| 1. Cut down on the amount of time you spend on work and other activities | | ○ | ○ | ○ | ○ | ○ | PRL01 |
| 1. Accomplish less than you would like | | ○ | ○ | ○ | ○ | ○ | PRL02 |
| 1. Were limited in the kind of work and other activities | | ○ | ○ | ○ | ○ | ○ | PRL03 |
| 1. Had difficulty performing the work or other activities (for example, it took extra effort) | | ○ | ○ | ○ | ○ | ○ | PRL04 |

**5 During the past 6 weeks, how much of the time have you had any of the following problems with your work or other regular daily activities as a result of any emotional problems (such as feeling depressed or anxious)?**

| *(emotional role limitation – ERL)* | All of the time | Most of the time | Some of the time | A little of the time | None of the time |  |
| --- | --- | --- | --- | --- | --- | --- |
| a. Cut down on the amount of time you spend on other activities | ○ | ○ | ○ | ○ | ○ | ERL01 |
| b. Accomplished less than you would like | ○ | ○ | ○ | ○ | ○ | ERL02 |
| c. Did work or other activities less carefully than usual | ○ | ○ | ○ | ○ | ○ | ERL03 |

**6 During the past 6 weeks, to what extent has your physical health or emotional problems interfered with your normal social activities, that is, your interpersonal relationships with family, friends, neighbours, or groups such as visits, meetings, wedding and burial ceremonies, etc?** *(social functioning – SF)*

| Not at all | Slightly | Moderately | Quite a bit | Extremely |  |
| --- | --- | --- | --- | --- | --- |
| ○ | ○ | ○ | ○ | ○ | SF01 |

**7 How much bodily pain have you had during the past 6 weeks?** *(bodily pain – BP)*

| None | Very mild | Mild | Moderate | Severe | Very severe |  |
| --- | --- | --- | --- | --- | --- | --- |
| ○ | ○ | ○ | ○ | ○ | ○ | BP01 |

**8 During the past 6 weeks, how much did pain interfere with your normal work (including both work outside the home and housework?** *(bodily pain – BP)*

| Not at all | A little bit | Moderately | Quite a bit | Extremely |  |
| --- | --- | --- | --- | --- | --- |
| ○ | ○ | ○ | ○ | ○ | BP02 |

**9 These questions are about how you feel and how things have been with you during the past 6 weeks. For each question, please give the one answer that comes closest to the way you have been feeling. How much of the time during the past 6 weeks …** *(vitality – VT/mental health – MH)*

|  | All of the time | Most of the time | Some of the time | A little of the time | None of the time |  |
| --- | --- | --- | --- | --- | --- | --- |
| a. Did you feel full of strength? | ○ | ○ | ○ | ○ | ○ | VT01 |
| b. Have you been very nervous? | ○ | ○ | ○ | ○ | ○ | MH01 |
| c. Have you felt so sad, hopeless, downhearted  and depressed that nothing could cheer you up? | ○ | ○ | ○ | ○ | ○ | MH02 |
| d. Have you felt calm and peaceful? | ○ | ○ | ○ | ○ | ○ | MH03 |
| e. Did you feel tired and worn out? | ○ | ○ | ○ | ○ | ○ | VT02 |
| f. Have you been happy? | ○ | ○ | ○ | ○ | ○ | MH04 |

**10 During the past 6 weeks, how much of the time has your physical health or emotional problems interfered with your social activities (like visiting friends, relatives, etc)?** *(social functioning – SF)*

| All of the time | Most of the time | Some of the time | A little of the time | None of the time |  |
| --- | --- | --- | --- | --- | --- |
| ○ | ○ | ○ | ○ | ○ | SF02 |

**11 How TRUE or FALSE is each of the following statements for you?** *(general health – GH)*

|  | Definitely true | Mostly true | Mostly false | Definitely false |  |
| --- | --- | --- | --- | --- | --- |
| 1. I seem to get sick a little more readily than other people | ○ | ○ | ○ | ○ | GH03 |
| 1. I am as healthy as anyone I know | ○ | ○ | ○ | ○ | GH04 |
| 1. I expect my health to get worse | ○ | ○ | ○ | ○ | GH05 |
| 1. My health is excellent | ○ | ○ | ○ | ○ | GH06 |

Thank you for completing these questions!
